# Sustainable Fire-Resistant Materials: Thermal, Physical, Mechanical, and Environmental Behavior of Walls with Waste from the Aquaculture Industry

**DOI:** 10.3390/ma18225086

**Published:** 2025-11-09

**Authors:** Begoña Peceño, Bernabé Alonso-Fariñas, Giovanna Vega, Daniel Carrizo, Carlos Leiva

**Affiliations:** 1 Escuela de Prevención de Riesgos y Medioambiente, Facultad de Ciencias del Mar, Universidad Católica del Norte, Larrondo 1281, Coquimbo 1781421, Chile; giovanna.vega@ucn.cl (G.V.); daniel.carrizo@ce.ucn.cl (D.C.); 2 Departamento de Ingeniería Química y Ambiental, Escuela Técnica Superior de Ingeniería, Universidad de Sevilla, Camino de los Descubrimientos s/n, 41092 Seville, Spain; bernabeaf@us.es (B.A.-F.); cleiva@us.es (C.L.)

**Keywords:** fireproof materials, seashell waste, semi-industrial scale, thermal properties, environmental properties

## Abstract

The aquaculture industry generates large amounts of shell waste, with limited recycling options at the industrial scale. This study explores the feasibility of substituting 20% of gypsum with seashell waste to produce sustainable, fire-resistant panels for non-load-bearing walls on a semi-industrial scale (2.4 × 2.2 × 0.1 m). The new composite exhibits high density (≈1500 kg/m^3^) and mechanical performance comparable to commercial gypsum. Thermal and fire tests confirmed its excellent insulation and stability: after 4 h of standard fire exposure, the non-exposed surface temperature remained below 80 °C, meeting European fire-resistance criteria. The incorporation of shell waste slightly reduced density and thermal conductivity (0.23 W/mK at 500 °C) without affecting strength or surface hardness. Environmental characterization revealed leaching and radionuclide levels well below regulatory limits, confirming its safety for building use. Overall, this work demonstrates, for the first time at a semi-industrial scale, the technical and environmental feasibility of reusing seashell waste as a gypsum substitute for fireproof materials. The proposed approach advances circular-economy strategies for aquaculture residues, providing an innovative pathway toward sustainable and low-impact construction products.

## 1. Introduction

The production system of the aquaculture industry is a heavy generator of waste. Shells make up between 40% and 70% of the weight of shellfish, despite the fact that the aquaculture business does not formally record the trash it produces [1]. It is estimated that just 25% of the eight million tons of aquaculture waste produced worldwide each year is recycled [2]. For example, the region of Taiwan and the country of South Korea generate approximately 160,000 metric tons of oyster shell waste [3] and 320,000 tons [4], respectively.

Despite having little to no market value, seashells are typically thrown in the trash or thrown into the ocean since there are no suitable alternatives [5]. A number of issues arise from the improper handling of this waste: (a) organic matter decomposition can cause the waste to smell [5], (b) the smells produced by the waste have an economic effect on the tourism industry [6], (c) illegal waste dumping introduces biological vectors like rats and mosquitoes into nearby populations [7], and (d) the lack of feasible and reasonably priced methods for handling shell waste has resulted in an increase in the unlawful disposal of waste near populated areas [8,9]. Neighbors’ complaints and protests have been sparked by the offensive smells and contamination from the dumping of waste [10,11,12]. For these reasons, the recycling of mollusk shells has been studied in different fields: (a) in aggregate in concrete [13,14,15,16,17,18], (b) as a pH regulator in acidic agricultural soils [18,19], (c) to replenish heavy metal-contaminated soils [20,21], (d) in poultry farming as a feed supplement for eggshell calcification [22,23], and (e) as a catalyst for biodiesel using CaO. Due to the large volume of natural resources required by the construction industry, the technical feasibility of using seashell waste has been assessed for different construction applications, such as in fine aggregates in concretes and mortars [24,25,26,27,28,29,30] and as a cement substitute [31,32]. Martínez-García et al. [33] evaluated the use of mussel shell waste as aggregates and mortars on a real scale. Nevertheless, environmental and economic studies related to recycling seashell waste have not demonstrated economic [6,30] nor environmental advantages as a substitute for limestone or aggregates [29,34,35]. As a result, there are few studies with a technology readiness level (TRL) higher than 5 (Technology basic validation in a laboratory environment) under controlled conditions, that can be scaled to real-world applications.

Passive fire protection is a barrier that stops the progression of smoke, prevents the spread of flames, contains thermal effects, and maintains the fire stability of structural elements for a specific time.

Gypsum has traditionally been the building material used for fireproofing. Gypsum experiences endothermic breakdown when heated, as in a fire, as a result of the following processes (1) and (2):

CaSO_4_∙2H_2_O→3/2H_2_O + CaSO_4_∙1/2 H_2_O (g) (1)

CaSO_4_∙1/2 H_2_O→1/2 H_2_O (g) + CaSO_4_ (2)

Semi-hydrate gypsum is produced at about 120 °C (Reaction (1)) [36]. At roughly 200 °C, the semi-hydrate gypsum transforms into anhydrous gypsum (reaction (2)) [37,38]. In total, 95% to 97% of seashells are made of calcium carbonate (CaCO_3_) [39,40] and traces of magnesium carbonate, silica, iron, and potassium oxides [41]. Just as with gypsum, shells at elevated temperatures (as in a fire) decompose endothermically (600 °C and 800 °C), as shown in Reaction (3):

*CaCO*_3_→*CaO* + *CO*_2_ (g) (3)

Different types of waste have been used in fire-resistant materials, which can be decomposed endothermally (phospho-gypsum [42], FGD gypsum [37], titanium gypsum [43], fluor-gypsum [44], or eggshells [45]). Others present a low thermal conductivity, such as coal fly ash [46], biomass fly ash [47], FeNi slag [48], ceramic stoneware [49], titanium dioxide waste [50], and recycled glass waste [51]. According to these investigations, certain waste may be employed in fire-resistant materials at doses ranging from 5 to 60 wt%.

The incorporation of waste materials into fire-resistant composites has been investigated at various scales [37,47,52,53,54,55]. At the laboratory level, studies have explored the recycling of seashell waste as a partial substitute for gypsum, such as the use of flue-gas-desulfurized gypsum combined with fly ash and titanium residues and the valorization of SiC sludge in geopolymeric matrices. However, few investigations have addressed these systems under real-scale or semi-industrial conditions. Leiva et al. [47] examined biomass ash as a raw material for large-scale fire-resistant panels, while Yang et al. [55] performed computational validation and in situ fire tests using recycled textile waste as a fire-retardant component.

Seashell waste as a substitute for gypsum has been shown to be technically and economically feasible and to reduce the carbon footprint [52,56,57]. This study analyses the mechanical, thermal, and fire resistance properties of seashell waste material on a semi-industrial scale to make progress on applications that can be used in different industries. To compare the performance of the new material on a semi-industrial scale, gypsum is used as the reference material, as it is the type of product most commonly used in non-load-bearing fire-resistant walls.

The purpose of this study is to evaluate the mechanical, physical, and fire insulation properties, as well as how its thermal properties (thermal conductivity, heat capacity) are affected by temperature changes on a semi-industrial scale. Based on international standards, the technical viability of seashells in fireproof materials has been assessed.

While previous studies have explored the partial substitution of gypsum with shell waste at the laboratory scale, most have remained limited to small samples and basic technical characterization. The novelty of this work lies in its scale-up to a semi-industrial prototype (2.4 × 2.2 × 0.1 m), enabling a realistic assessment of its mechanical, thermal, and fire-resistance behavior under standardized conditions. Moreover, the study incorporated an environmental risk evaluation, including leaching and radionuclide activity, that has not been previously addressed for shell-based fireproof composites. Together, these aspects provide a comprehensive validation of the material’s technical and environmental feasibility for potential industrial implementation, advancing the technology readiness level (TRL) of seashell-gypsum composites.

## 2. Materials and Methods

### 2.1. Materials

**Figure 1:**
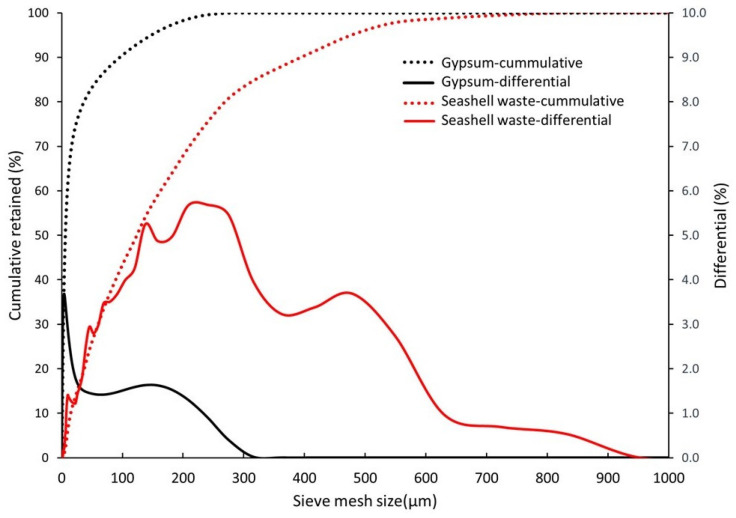
Granulometric distribution of raw materials used.

We utilized waste seashells from “Mesodesma donacium” (SW), a species of surf clam, obtained from a canning facility in La Serena, Chile. To remove organic matter and salts, the shell waste was washed using a water-to-waste ratio of 1.5 L/kg. Subsequently, it was thermally treated at 300 °C for 24 h to eliminate the organic matter and then ground and sieved as shown in Figure 1 [52]. A sample of commercial gypsum material (G) that complies with the EN 13279-1 standards was used without further processing [58].

For both seashell and gypsum samples, chemical compositions were measured with an absorption spectrometer in compliance with ASTM D 3682 [59] for major elements (see Table 1) after heating to 750 °C for 3 h.

In shell waste, CaCO_3_, also known as CaO, is the principal compound. Reaction (3) yields CO_2_ and CaO at 650 °C. The latter is represented as loss on ignition (LOI) in Table 1. In the seashell waste (SW), the CaCO_3_ composition was 99%. Gypsum (G) is primarily composed of CaSO_4_. Specific gravity was determined according to ASTM D 854 [60]. The specific gravity was similar in both raw materials. The materials’ particle size distribution is displayed in Figure 1. Seashell waste has an average particle size 20 times larger than traditional gypsum (G) (130.0 μm for SW vs. 6.6 μm for G). The seashell waste presented higher differential percentages (between 25 and 600 μm) than gypsum (between 0.3 and 240 μm).

After being calcined, the seashells underwent additional processing, which involved crushing them with a jaw crusher until their particle size was reduced to less than 0.6 mm.

#### Sample Fabrication Method

The specimens of the product were manufactured following standard laboratory conditions at a temperature of 25 °C and 45% humidity. Sample preparation involved weighing and blending raw materials, namely gypsum and seashell waste, using a blender. Two compositions were created: one with 20% seashell waste and 80% traditional gypsum (designated as 20SW), and another with 100% traditional gypsum (labeled as 100G). The substitution ratio of 20% shell waste was selected according to previous optimization studies carried out at the laboratory scale [61]. In that work, substitution levels between 5% and 40% were evaluated, identifying 20% as the optimal composition. Water was then added. As seen in other products with comparable qualities, a constant water-to-solid ratio of 0.40 was maintained in the mixture [52]. After 24 h, all materials were demolded and subjected to curing at room temperature, averaging 20 °C, with consistent humidity maintained at an average of 45% relative humidity for an additional period of 27 days.

### 2.2. Methods

#### 2.2.1. Chemical and Physical Properties

The thermogravimetric analysis (TG) was performed according to Li et al. [62].

For each composition (20SW and 100G), the porosity and distribution of pore sizes in the range of 1 to 10,000 nm were assessed using high-pressure mercury intrusion porosimetry (MIP). For this, the following settings were used: 140° contact angle, 480 mN/m surface tension, and 413 MPa maximum pressure.

Density (d) and moisture content (W) were in compliance with ASTM E 605 [63] and EN 12859 [64], respectively. Each physical test was repeated three times for each composition. The results displayed are the average of the outcomes from each analysis. Using 34 mm diameter and 40 mm height cylindrical molds, the physical properties were ascertained.

#### 2.2.2. Mechanical Properties

The compression strength was assessed using specimens that had dimensions of 4 cm across three dimensions (width, length, and height). To determine the flexural strength (SF), prismatic samples were utilized. The dimensions of the samples were 10 cm, 4 cm, and 4 cm in length, width, and height, respectively. A machine for compression testing was employed to assess the compressive and flexural strengths of 20SW and 100G in compliance with EN 13279-2 [65]. Each mechanical test was repeated three times for each composition. The results displayed are the average of the outcomes from each analysis.

The surface hardness (SH) was measured using a Shore C durometer in accordance with EN 13279-2 [65]. Five surface hardness tests were conducted for each face of the specimen, and the reported results are the averages obtained from these analyses. Values with a dispersion greater than 10% were excluded from the average calculation, since such deviations are generally associated with experimental errors caused by the non-perpendicular positioning or variable pressure applied during the durometer measurement [66].

Impact resistance (IR) was measured according to EN 520 [67], using a 24 × 24 cm by 3 cm thick panel. A ball of steel with 245j potential energy was used to strike the panel, and the diameter of the impression it left behind (in millimeters) was measured. The test was repeated three times, and the average results are shown.

#### 2.2.3. Thermal Properties

The assessment of specific heat was conducted according to the method outlined by Leiva et al. [47], using differential scanning calorimetry (DSC). For this, 10 °C per minute of heating [62] was used for the DSC measurements, which covered a temperature range of 20 to 500 °C.

Thermal conductivity was measured in accordance with ASTM E1461-13 [68]. Thermal diffusivity and density were determined via the flash method using an instrument from Linseis GmbH (Selb, Germany). Thermal diffusivity was measured from 20 to 500 °C, and thermal conductivity was determined by Equation (4):

λ(T) = Cp(T)∙a(T)∙ρ(T)(4)

where λ(T), Cp(T), a(T), and ρ(T) were thermal conductivity (W/mK), specific heat (J/gK), thermal diffusivity (m^2^/s), and density (g/m^3^), respectively.

#### 2.2.4. Fire Insulating Capacity

**Figure 2:**
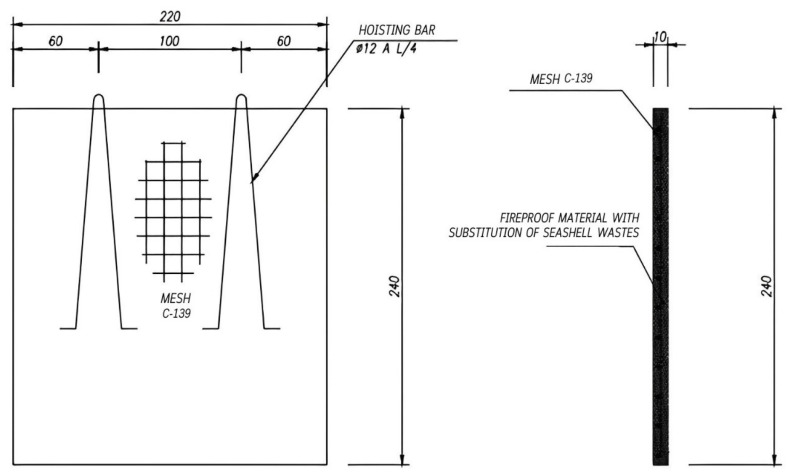
Prototype design on a semi-industrial scale.

Likewise, a 2.2 × 2.4 m by 10 cm thick wall was built for 20SW. In addition, a “C-139” mesh type reinforcement was incorporated in the center of the wall to avoid the risk of failure during the transfer and lifting of the wall between laboratories (Figure 2). The fresh mixture was homogenized using a mechanical blender at 600 rpm for 10 min and compacted on a vibrating table (50 Hz, 3 min) to ensure uniform density and eliminate air voids. The panel was cured under controlled conditions (20 ± 2 °C, 45 ± 5% RH) for 28 days.

Following the guidelines of the European Standard [69], the temperature of the furnace was regulated to conform to the requirements for a standard fire (Equation (5)).

T = 20 + 345log(1 + 8t)(5)

where t represents the time in minutes, and T stands for the fire temperature in °C.

**Figure 3:**
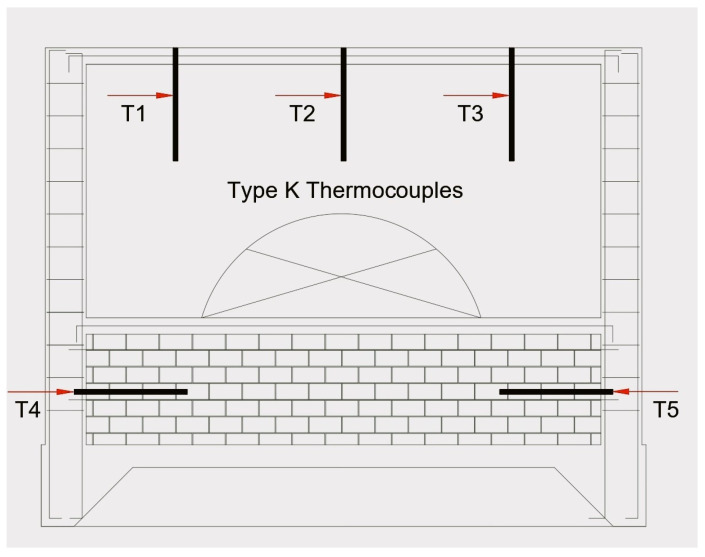
Test furnace scheme.

The examination took place at IDIEM’s fire test laboratory in Santiago, Chile. The facility features a testing furnace outfitted with a modulating gas burner boasting a thermal power rating of 1700 kW. The furnace’s opening has dimensions of 2.2 m in width and 2.4 m in height. Surrounding the furnace wall is a “Durablanket” ceramic blanket (density: 96 kg/m^3^) and a layer of gypsum, creating an airtight seal around the entire outer perimeter to prevent heat transfer through the wall’s sides. The monitoring of its temperature was carried out using five type K thermocouples, each with a 1.6 mm thick wire (see Figure 3). The furnace’s operation was managed through computerized control. The temperature of the non-exposed face was measured with an Extech Data Logger Infrared Thermometer (model 42545, series I0096007) (Flir, Wilsonville, OR, USA) with a range of −50 to 1000 °C.

#### 2.2.5. Environmental Risk Assessment

– Leaching test

The fresh leaching solution, consisting of pH 7 water, underwent eight consecutive alterations in accordance with the NEN 7375 [70] test protocol. This testing methodology is deemed reasonably analogous to natural rainfall, commonly regarded as a primary leaching solution for outdoor building materials. The metal content in the leachates was assessed using inductively coupled plasma atomic emission spectroscopy.

– Radionuclide test

Construction materials and waste may include radioactive nuclides that exist naturally [71], categorizing them as substances with inherent radioactivity [72]. As such, a test was performed to assess radioisotope activity. The material containing shell waste and the traditional gypsum counterpart underwent size reduction through a ball mill process. Subsequently, the finely ground material was placed in a polystyrene 80 cm^3^ Petri dish and enclosed under vacuum to minimize the release of 222-Rn. To detect primary gamma rays, we utilized a Canberra coaxial detector with low background radiation (GR-6022) featuring a reverse electrode made of high-purity germanium (HPGe). This detector had a volume of 180 cm^3^, with respective energy levels of 122 keV and 1332 keV, displayed a resolution of 1.4 keV and 2.3 keV, and was positioned securely in a lead shielding (thick: 10 cm) with low radioactivity. Its relative efficiency was 60%.

Additionally, Equation (6), which determines the activity concentration (Bq/kg) of the primary naturally occurring radionuclides in the building materials (including K-40, Th-232, and Ra-226), was used to measure the activity concentration index (ACI) [71].

ACI = (CK/3000) + (CTh/200) + (CRa/300)(6)

where CK, CTh, and CRa represent the activity concentrations of K-40, Th-232, and Ra-226, respectively. The activity concentration is measured in Bq/kg.

## 3. Results

### 3.1. Chemical and Physical Properties

**Figure 4:**
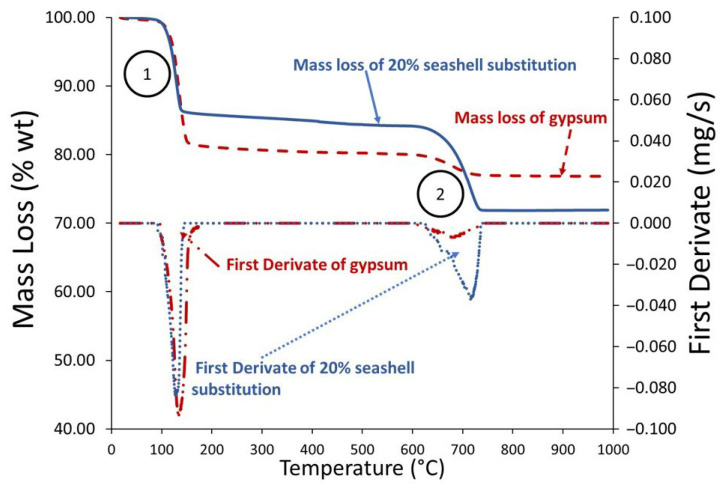
TG first derivative (mg/s) and mass loss (wt%) of gypsum partially substituted (20 wt%) with seashell waste (20SW) and gypsum (100G).

As shown in Figure 4, the mass of both materials (20SW and 100G) began to decrease between 100 and 150 °C (point 1 in Figure 4), reaching the maximum rate of mass loss at approximately 139 °C, as indicated by the first derivative (DTG) curves. The first derivative represents the rate of weight change with respect to temperature (dm/dT), which highlights the temperatures at which the most intense decomposition or dehydration reactions occur. In this case, the peak in the first derivative curve corresponds to the temperature where the removal of chemically bound water from gypsum is most pronounced. This initial reduction is associated with the dehydration of calcium sulfate dihydrate (CaSO_4_·2H_2_O), which progressively transforms into calcium sulfate hemihydrate (CaSO_4_·0.5H_2_O), and then into anhydrous calcium sulfate (CaSO_4_), according to reactions (1) and (2) [37,38]. Due to the relatively high heating rate (10 °C/min), these two endothermic reactions overlap, resulting in a single broad mass loss step [73]. For 20SW, which contains approximately 80% gypsum, the weight loss was proportional to the CaSO_4_·2H_2_O content, about 13% for 20SW and 18% for 100G.

At higher temperatures, between 600 °C and 735 °C (point 2 in Figure 4), a second endothermic peak was observed, corresponding to the decomposition of calcium carbonate (Reaction (3)). The first derivative curve shows the maximum reaction rate near 700 °C, confirming the decarbonation process described in previous studies [74]. This reaction caused a mass loss of approximately 12% in 20SW and 3% in commercial gypsum (100G), consistent with their different calcium carbonate contents (see Table 1).

**Figure 5:**
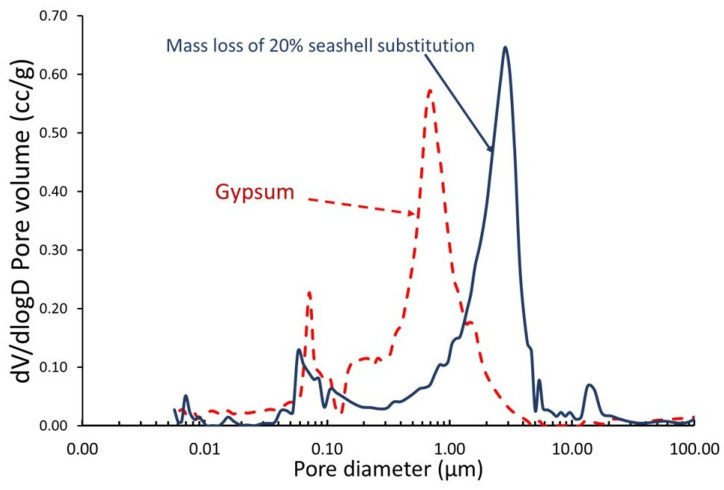
MIP of gypsum and gypsum partially substituted (20 wt%) with seashell waste.

As shown in Figure 5, the use of 20% seashell waste (20SW) produced, primarily, pore sizes of 3 µm and secondarily, pore dimensions ranging from 0.05 µm to 15 µm. However, in the case of commercial gypsum (100G), pore sizes of 1 µm are produced. The pore size of gypsum with seashell waste is similar to other studies where limestone and gypsum are used in compositions with similar particle sizes [75].

The incorporation of 20% seashell waste did not cause substantial variations in the moisture with respect to 100G (see Table 2).

The incorporation of 20% of seashell waste caused a slightly lower density (a decrease of 2%) (see Table 2). This is because (1) the specific gravity of gypsum was slightly higher (Table 1) than that of seashell waste and (2) the seashell waste exhibited a larger particle size (as depicted in Figure 1), which generated a larger pore size (Figure 5). According to EN 12859, both 20SW and 100G were categorized as high-density due to both materials exceeding >1100 kg/m^3^ [64].

### 3.2. Mechanical Properties

The incorporation of 20% seashell waste did not cause variations in the surface hardness in comparison with 100G (Table 3). Since both 100G and 20SW were classified as high-density, both materials were required to have a surface hardness exceeding 80 Shore C [64].

The 20SW had an impact resistance of 1.5 ± 0.07 cm, which was marginally less than that of gypsum. This value is in line with the literature, because it is marginally less than the 2.1 cm value displayed by commercial gypsum [58].

The 20SW materials exhibited a non-significant decrease in both compression and flexural strength compared to the 100G materials. Gypsum panels must have a flexural strength of at least 0.83 MPa and a compressive strength of at least 2.0 MPa, in accordance with EN 13279-1 [58]. As a result, every mortar that was produced met this requirement. Both the flexural and compressive mechanical properties are similar to those of other studies [76] and to other materials using automotive wastes [77].

### 3.3. Thermal Properties

**Figure 6:**
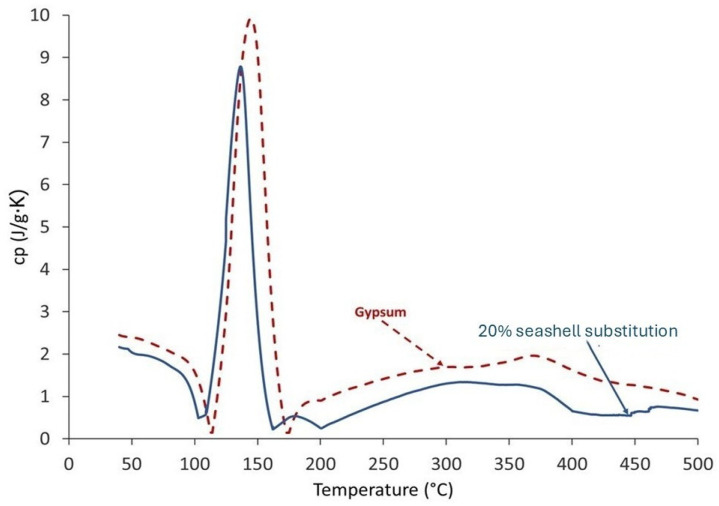
Specific heat capacity of gypsum (100G) and gypsum partially substituted (20 wt%) with seashell waste (20SW).

Heat capacity is the ability of a material to absorb heat at different temperatures. The 20SW showed less heat capacity than the gypsum (see Figure 6). This can be observed between 50 and 100 °C, where the heat capacity of 20SW was lower than 100G, due to its lower humidity (see Table 2). Note that at 50 °C, the peak began to decrease due to the moisture content in the mortars. At 100 °C, the heat capacity exhibited a higher peak due to the evaporation of the water chemically bonded to gypsum (Reactions (1) and (2)) [78]. In the 20SW, the maximum height of the peak was lower than 100G due to the lower gypsum content. In the gypsum, after 200 °C and until the end of the test, the increase in heat capacity was less than 5%. A similar trend was observed for the material with 20% seashell waste, except for an increase at 450 °C due to carbonate decomposition (Reaction (3)) [77].

**Figure 7:**
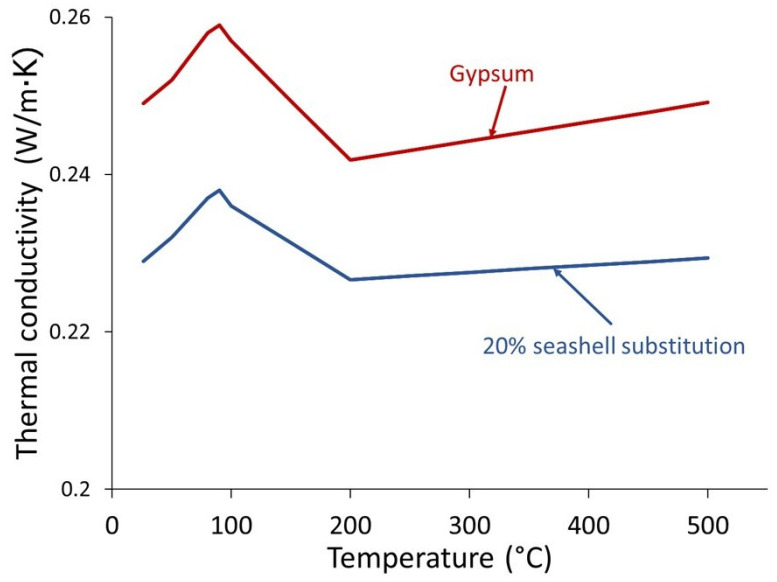
Thermal conductivity of gypsum (100G) and gypsum partially substituted (20 wt%) with seashell waste.

As shown in Figure 7, the conductivity of the material composed of 20% seashell waste (20SW) was less than that of the 100% gypsum material (100G). This was because the material composed of 20% seashell waste had a greater quantity of pores (see Figure 5) due to having a lower density (Table 2). According to EN 12859 [64], for high-density materials, the thermal conductivity must be less than 0.56 W/mK at 23 °C. At around 100 °C, the conductivity began to decrease until 200 °C, due to the evaporation of water according to the TG (Figure 4). Between 100 °C and 200 °C, 100G had a steeper slope than 20SW because 100G had a greater amount of water (Table 2). After 200 °C, both samples increased their thermal conductivity. However, 100G had a smaller slope than 20SW because the evaporation of water generated an increase in porosity and therefore a reduction in density [38].

### 3.4. Fire Resistance

**Figure 8:**
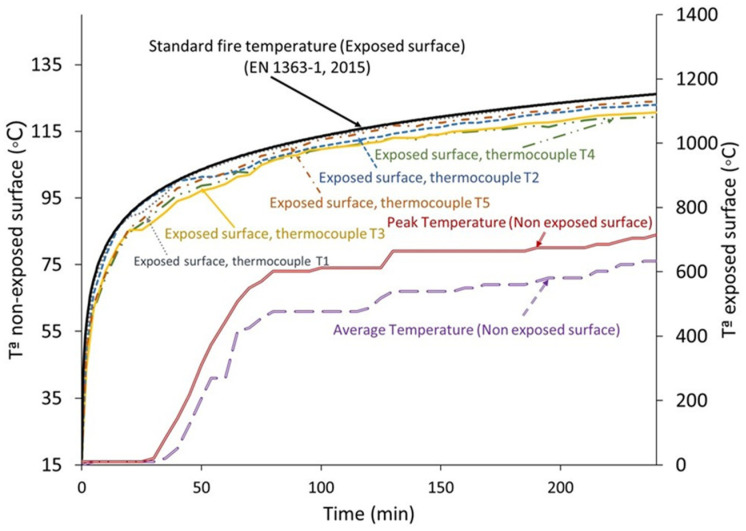
Time–temperature ratio of gypsum partially substituted (20 wt%) with seashell waste [69].

In relation to European Standard EN 1363, Figure 8 depicts the temperature curve with respect to time on the prototype’s non-exposed surface (20SW) [69].

The temperature profile of the unexposed surface remains relatively constant at approximately 20 °C during the initial forty minutes and then exhibits a linear ascent until reaching 61 °C, marking the onset of the evaporation plateau. Within this plateau, two distinct periods emerge: the first phase (1) is attributed to the moisture evaporation within the material, augmenting the specific heat capacity, and spans until minute 120; the second phase (2) is identified by the liberation of water that is chemically bound within the material (carbonates and gypsum), manifesting at around 79 °C and persisting until the conclusion of the test. The temperature remains below 100 °C due to the saturation water pressure of 1 atm and is influenced by the thermal equilibrium (radiation and convection) established between the unexposed surface and the surrounding environment [37]. The duration of the evaporation plateau is strongly dependent on the material’s porosity [79]. High pore connectivity facilitates the diffusion of water vapor, preventing its accumulation within the pore network and enhancing the dehydration rate [80]. Conversely, in less porous materials, water vapor may become trapped within the matrix, leading to an increase in internal vapor pressure, which partially inhibits dehydration and results in a temperature rise within the system [78].

**Figure 9:**
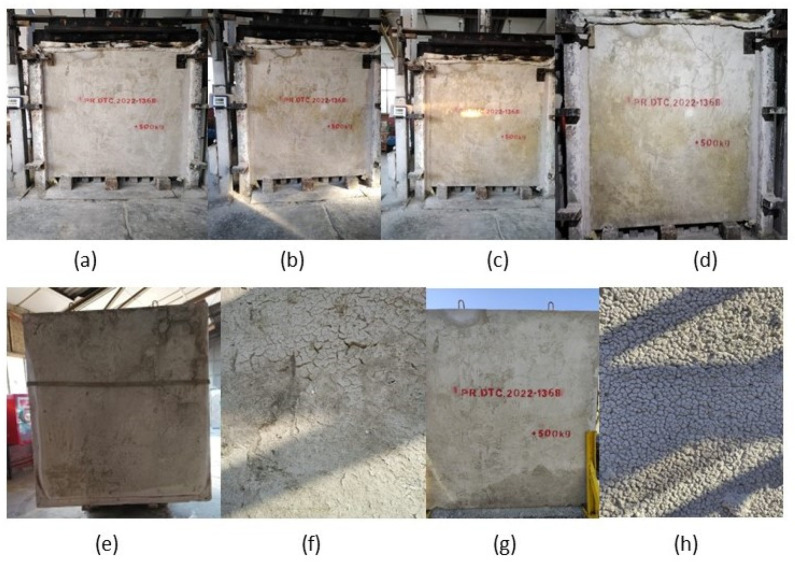
Visual photographs of the material with 20% seashell waste at various stages. (**a**) non-exposed surface at 0 h, (**b**) 1.5 h, (**c**) 3 h, (**d**) 4 h, (**e**) exposed surface the next day, (**f**) detail of the exposed face the next day, (**g**) non-exposed surface after a month, (**h**) exposed surface after a month.

Throughout the fire test, no observable gas emissions occurred, and mechanical stability was sustained, as depicted in Figure 9a–d.

The thickness of the material also affects the duration of the evaporation plateau. When the material is doubled in thickness, the duration time of the evaporation plateau is multiplied by eight [53]. Therefore, reducing the wall thickness to 5 cm would result in the plateau lasting approximately 87 min.

The 5 cm wall thickness shows a performance similar to that of commercial products, given that, in comparison, commercial rock-wool panels with 50 mm thickness typically exhibit fire-resistance ratings between 60 and 90 min [81,82,83]. In the case of 20 cm gypsum-based walls, commercial suppliers report fire-resistance behavior comparable to that achieved with a 20% partial substitution of gypsum by seashell waste [84].

As shown in Figure 9h, the surface exposed to fire suffered spalling that was 5 cm thick. This was due to the formation of microcracks (Figure 9f) caused by the loss of surface tension in the material as a consequence of mechanical stresses induced by the steam overpressure inside the wall [85,86]. The non-exposed surface did not suffer any alterations (Figure 9g).

### 3.5. Environmental Risk Assessment

#### 3.5.1. Leaching Test

The Dutch Soil Quality Decree’s (DSQD) standards and the leaching test results were compared [87]. These regulations were created to stop building materials from contaminating the land and surface waters. The leaching results obtained for the gypsum and the 20% seashell waste material were below the DSQD limits in all cases (see Table 4). The results demonstrate that both pure gypsum (100G) and the mixture containing 20% seashell waste (20SW) exhibit extremely low leaching values for all analyzed elements, fully complying with the limits established by the DSQD regulation. In most cases, the leached concentrations represent less than 1% of the maximum allowable value, and even smaller fractions for several heavy metals, indicating a wide environmental safety margin. For example, the zinc leached from the 100G sample is approximately 0.2 mg/m^2^, equivalent to only 0.03% of the regulatory limit. In the case of sulfates, the partial substitution of gypsum with 20% seashell waste (20SW) resulted in a reduction of about 20% compared to the 100G material, consistent with the lower proportion of gypsum present. The obtained values remain far below the DSQD threshold of 165,000 mg/m^2^. This decrease suggests that the incorporation of seashell residues not only does not increase the contaminant load but may also contribute to a slight environmental improvement by reducing sulfate release compared to conventional materials.

#### 3.5.2. Radionuclide Test

The European Directive 2013/59/EURATOM [88] establishes 1.0 mSv/year as the permissible gamma radiation level for prolonged exposure. For gamma radiation to be less than 1 mSv/year, the ACI must be below 1 [79]. For 20SW, it demonstrated half the Ra-226, as the seashell waste did not contain RA-226 (see Table 5). The activity and the ACI were less than 1 for both materials (100G and 20SW).

## 4. Conclusions

The purpose of this study was to demonstrate the feasibility of seashell waste as a substitute for gypsum in non-load-bearing fire-resistant walls. The findings of a semi-industrial scale study on the material’s mechanical and thermal behavior are reported. The following useful conclusions can be drawn from this research regarding the technical feasibility of recycling seashell waste into this type of material:

– Substituting gypsum with seashell waste does not cause a large change in the bulk density of the material. The bulk density of the new material is 1.8% less than that of gypsum.
– The use of seashell waste in place of gypsum has no mechanical disadvantages. When compared to traditional materials, the material made with seashell waste exhibits comparable mechanical properties.
– A 20% substitution of seashell waste for gypsum has a negligible effect on the material’s thermal properties. At 500 °C, the new material’s thermal conductivity decreased to 0.23 W/mK compared to 0.25 W/mK for gypsum. Similarly, at room temperature (20 °C), both materials have comparable thermal conductivities.
– In terms of fire resistance, because of a reduction in water retention capacity, replacing 20% of the gypsum with seashell waste shortens the evaporation plateau’s duration. In contrast to the conventional material, the material containing 20% seashell waste has a lower slope after the evaporation plateau. Therefore, the addition of 20% seashell waste does not affect the fire resistance properties of the material. Additionally, the tested material’s mechanical qualities are satisfactory; during the fire resistance test, it exhibited no discernible crumbling, cracking, or deformation.
– Regarding the environmental properties, the incorporation of 20% shell waste in gypsum-based materials does not affect the material’s potential for leaching nor its radiological behavior.

Furthermore, this study enhances the technology readiness level (TRL) of the developed composite material by demonstrating its effectiveness at a pilot scale under near-industrial conditions. The results confirm that gypsum partially substituted (20 wt%) with seashell waste can achieve comparable fire and mechanical performance to commercial gypsum-based materials while maintaining environmental safety. This advancement not only validates the technical feasibility of the process but also reinforces its alignment with circular economy principles, transforming abundant marine shell waste into a valuable raw material for sustainable construction. Future research should focus on the transition toward full industrial-scale production, long-term durability assessment under moisture and thermal cycling, and the validation of these composites in load-bearing structural applications to consolidate their use in safe, resilient, and environmentally responsible buildings.

## 5. Patents

From this study emerged the patent WO2023092246A1: Aquaculture waste ecopanel having passive fire protection properties and compressive and flexural strength.

## Figures and Tables

**Table 1:** Specific gravity (g/cm^3^), average particle size (µm), and chemical composition (wt%).

	SW ^2^	G ^3^
SiO_2_ (wt%)	0.3	0.9
SO_3_ (wt%)	0.2	45.7
Al_2_O_3_ (wt%)	N.D. ^4^	0.2
CaO (wt%)	54.6	40.6
Na_2_O (wt%)	0.9	0.1
MnO (wt%)	0.0	0.0
LOI ^1^ (wt%)	44.0	12.5
Median size (μm)	130.0	6.6
Specific gravity (g/cm^3^)	2.6	2.9

Note: ^1^. LOI for loss on ignition; ^2^. SW stands for shell waste from clams; ^3^. G for gypsum; and ^4^. N.D. for non-detectable. In addition, the concentrations of MgO, K_2_O, TiO_2_, P_2_O_5_, and Fe_2_O_3_ were measured, and they were found to be non-detectable in both samples.

**Table 2:** Physical properties of gypsum (100G) and gypsum partially substituted (20 wt%) with seashell waste (20SW).

	W ^1^ (%)	D ^2^ (kg/m^3^)
100G	17 ± 1	1520 ± 2
20SW	15 ± 1	1492 ± 17

Note: ^1^. W for moisture content, ^2^. D for Density.

**Table 3:** Mechanical properties of gypsum (100G) and gypsum partially substituted (20 wt%) with seashell waste (20SW).

	SH ^1^ (Shore C)	IR ^2^ (cm)	Sc ^3^ (MPa)	Sf ^4^ (MPa)
100G	94 ± 1	2.1 ± 0.1	14.8 ± 1.4	4.0 ± 0.8
20SW	94 ± 2	1.5 ± 0.1	14.7 ± 2.3	4.0 ± 0.6

Note: ^1^ SH for surface hardness, ^2^ IR for impact resistance, ^3^ Sc for Compression strength, ^4^ Sf for Flexural strength.

**Table 4:** NEN 7345 leaching test result for gypsum (100G) and gypsum partially substituted (20 wt%) with seashell waste (20SW) compared with DSQD limits.

	100G (mg/m^2^)	20SW (mg/m^2^)	DSQD Limits (mg/m^2^)
Se	4	3.5	4.8
Hg	0.7	0.7	1.4
Sn	2.5	2.4	50
Ba	3.7	2.3	1500
Pb	3.5	1.2	400
Sb	2.6	2.2	8.7
Cd	0.7	0.7	3.8
Co	0.2	0.2	60
V	3.8	3.8	320
Cr	0.2	0.2	120
As	5.2	5	260
Mo	2.4	1.6	144
Ni	2.8	1.4	81
Zn	9	6	800
Cu	1.2	1.2	98

**Table 5:** Activity concentrations and ACI values of gypsum (100G) and gypsum partially substituted (20 wt%) with seashell waste (20SW).

	100G (mg/m^2^)	20SW (mg/m^2^)
K-40 (Bq/kg)	MNDA (1)	MNDA (1)
Th-232 (Bq/kg)	MNDA (1)	MNDA (1)
Ra-226 (Bq/kg)	3.6 ± 1.1	1.8 ± 0.7
ACI	0.012	0.006

## Data Availability

The original contributions presented in this study are included in the article. Further inquiries can be directed to the corresponding author.
